# Bacterial Bio-Resources for Remediation of Hexachlorocyclohexane

**DOI:** 10.3390/ijms131115086

**Published:** 2012-11-15

**Authors:** Analía Alvarez, Claudia S. Benimeli, Juliana M. Saez, María S. Fuentes, Sergio A. Cuozzo, Marta A. Polti, María J. Amoroso

**Affiliations:** 1 Pilot Plant of Industrial and Microbiological Processes (PROIMI), CONICET, Av. Belgrano y Pasaje Caseros, 4000 Tucumán, Argentina; E-Mails: alvanalia@gmail.com (A.A.); cbenimeli@yahoo.com.ar (C.S.B.); jsaez@proimi.org.ar (J.M.S.); soledadfs@gmail.com (M.S.F.); sergio.cuozzo@gmail.com (S.A.C.); mpolti@proimi.org.ar (M.A.P.); 2 Natural Sciences College and Miguel Lillo Institute, National University of Tucumán, Miguel Lillo 205, 4000 Tucumán, Argentina; 3 North University of Saint Thomas Aquines, 9 de Julio 165, 4000 Tucumán, Argentina; 4 Biochemistry, Chemistry and Pharmacy College, National University of Tucumán, Ayacucho 471, 4000 Tucumán, Argentina

**Keywords:** organochlorine pesticides, γ-hexachlorocyclohexane, bioremediation, *Streptomyces* genus

## Abstract

In the last few decades, highly toxic organic compounds like the organochlorine pesticide (OP) hexachlorocyclohexane (HCH) have been released into the environment. All HCH isomers are acutely toxic to mammals. Although nowadays its use is restricted or completely banned in most countries, it continues posing serious environmental and health concerns. Since HCH toxicity is well known, it is imperative to develop methods to remove it from the environment. Bioremediation technologies, which use microorganisms and/or plants to degrade toxic contaminants, have become the focus of interest. Microorganisms play a significant role in the transformation and degradation of xenobiotic compounds. Many Gram-negative bacteria have been reported to have metabolic abilities to attack HCH. For instance, several *Sphingomonas* strains have been reported to degrade the pesticide. On the other hand, among Gram-positive microorganisms, actinobacteria have a great potential for biodegradation of organic and inorganic toxic compounds. This review compiles and updates the information available on bacterial removal of HCH, particularly by *Streptomyces* strains, a prolific genus of actinobacteria. A brief account on the persistence and deleterious effects of these pollutant chemical is also given.

## 1. Introduction

Highly toxic organic compounds have been synthesized and released into the environment for direct or indirect application over a long period of time. Persistent organic pollutants (POPs) such as pesticides, fuels, polycyclic aromatic hydrocarbons (PAHs), polychlorinated biphenyls (PCBs), chlorophenols and dyes are some of these types of compounds [1]. POPs are extremely resistant to biodegradation by native flora [2] compared to the naturally occurring organic compounds that are readily degraded upon introduction into the environment. Therefore, hazardous wastes and chemicals have become one of the greatest worldwide problems of modern society. Among POPs, organochlorine pesticides (OPs) constitute a major environmental problem, because of their high toxicity, persistence in the environment and ability to bioaccumulate in the food chain [3–5]. These compounds reach aquatic environments through effluent release, atmospheric deposition and runoff among other ways [6]. Because of the low water solubility, OPs have a strong affinity for particulate matter, and consequently, sediments can serve as an ultimate sink [7,8].

**Figure 1:**
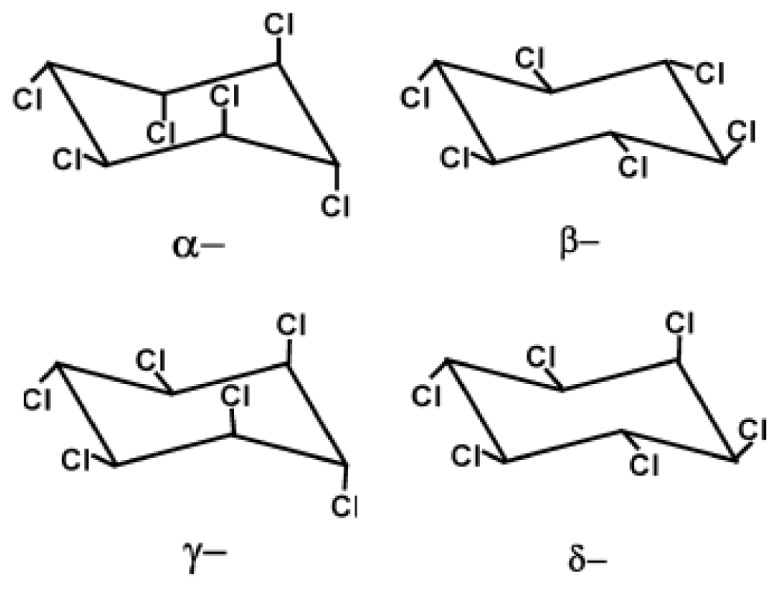
Structures of hexachlorocyclohexane isomers (adapted from Manickam *et al.*[11]).

The organochlorine compound 1,2,3,4,5,6 hexachlorocyclohexane (HCH) is a broad-spectrum pesticide that was used on a large scale worldwide since the 1940s, and is available in two formulations: technical-grade HCH (a mixture of different isomers, mainly α-(60%–70%), β-(5%–12%), γ-(10%–15%) and δ-HCH (6%–10%)) and lindane (almost pure γ-HCH) (Figure 1). This mixture was marketed as an inexpensive insecticide, but since γ-HCH is the only isomer that exhibits strong insecticidal properties, it has been common to refine it from technical-grade HCH and market it under the name “lindane”. As a result, large quantities of byproducts (mixtures of other HCH isomers) were dumped around production facilities leading to the contamination of many sites worldwide: For each ton of lindane produced, 8–12 tons of wastes are generated (generally enriched in the α and β isomers) [9]. Accordingly, there are very few officially recognized OP-contaminated sites in South America, and these are located principally in heavily populated industrial areas, *i.e.*, Sao Paulo (Brazil), Buenos Aires (Argentina), and Santiago and Concepción (Chile). However, these official numbers grossly underestimate the real situation because of the existence of illegally contaminated sites [10]. For instance, the most important known illegal disposal of more than 30 tons of HCHs and other organochlorines such as DDT, took place in the small town of Argentina, in the province of Santiago del Estero [5,10].

All HCH isomers are acutely toxic to mammals, due to their mutagenic, teratogenic and carcinogenic properties [12]. Although nowadays its use is restricted or completely banned in most countries, it continues posing serious environmental and health concerns [13]. This is because γ-HCH was widely used as insecticide, which added to its high environmental persistence, ensuring lindane residues would be found all over the world and reaching aquatic environments through effluent release, atmospheric deposition and runoff, among other ways [6,12]. In this context, residues of γ-HCH and others HCH isomers have now been reported by many countries [13] in samples of air [14,15], water [16,17], soil [17–20], food commodities [21–23], milk [24,25] and even in human blood [26] and adipose tissue [27]. Since HCH spread to remote areas due to migration via various routes, their residues have been detected in Arctic, Antarctic and Pacific Ocean [28], although they had never been used or produced there. The recent inclusion of the α, β and γ-HCH isomers in the Stockholm Convention list of POPs has led to a renewed interest in these contaminants and the remediation of affected sites.

Traditional technologies routinely used for remediation of contaminated environmental soil include excavation, transport to specialized landfills, incineration, stabilization and vitrification. However, bioremediation technologies, which imply microorganisms and/or plants to degrade toxic contaminants in soil into less toxic and/or nontoxic substances, have become the focus of interest [29]. Microbial degradation is regarded as an important process of Ops-removal from sediments, and to date, many researchers have studied this issue [2]. In particular, aerobic and anaerobic bacteria are the most active agents of bioremediation [30], having the ability to interact, both chemically and physically, with substances leading to structural changes or complete degradation at the target molecule, playing a significant role in the transformation and degradation of pesticides. Even the most persistent pesticides can be metabolized to some extent by microbial cultures, either by utilization of the compounds as a source of energy or nutrients, or by cometabolism with other substrates supporting microbial growth. This last process is probably the most widespread mechanism for pesticide biodegradation [31]. Complete mineralization of pesticides or their transformation to nontoxic products is desirable, but it is more likely to be carried out by consortia of microorganisms than by single isolates [32–34].

As a way of not altering natural ecosystems, the trend in the last years has been the search and utilization of native bacteria. Many Gram-negative bacteria have been reported to have metabolic abilities to attack lindane. Several bacterial strains (predominantly *Sphingomonas* strains) capable of degrading HCH isomers under aerobic conditions have been isolated from contaminated soils [35–38]. On the other hand, among Gram-positive microorganisms, actinobacteria—particularly those belonging to the *Streptomyces* genus—have a great potential for biodegradation of organic and inorganic toxic compounds [39,40]. Benimeli *et al.*[41–45], Cuozzo *et al.*[46,47], Fuentes *et al.*[5,48] and Saez *et al.*[34] isolated and selected wild-type *Streptomyces* spp. which were able to tolerate and remove several OPs from culture media and soils.

Regarding eukaryotic cells, fungi have great capacity to degrade xenobiotic. Among them, white-rot fungi have been widely used to degrade several classes of pesticides (including γ-HCH) due to their ligninolytic potential [49–52]. Likewise, many plants—natural, transgenic, and/or associated to rhizosphere microorganisms—are extraordinarily active in removing or immobilizing pollutants, and particularly HCH isomers [38,53–55]. However, this article is focused on bacteria, although the HCH-bioremediation capacity of certain plants associated with microorganisms is mentioned.

In this review, it is intended to compile and update the information available on bacterial degradation of HCH. A brief account on the persistence and deleterious effects of these pollutant chemicals is also given.

## 2. Physicochemical Properties of Hexachlorocyclohexane: Persistence and Deleterious Effects

Persistence and mobility of pesticides are defined primarily by their physical and chemical properties [37,56]. Li and Wania [57] compiled selected physicochemical properties, including vapor pressure, water solubility, Henry’s law constant (*H*), octanol-water partition coefficient (*K*_OW_), and octanol-air partition coefficient (KOA) of major OPs. These properties have been used to screen many chemicals for persistent organic pollutants criteria [58]. For HCH isomers, such properties are quite different from one to another, depending on the chlorine positions in the cyclohexane ring (Figure 1) [12,37]. For instance, the vapor pressure of α-HCH is somewhat less than of γ-HCH. α-HCH has also been shown to be slightly more lipophilic than γ-HCH (log *K*_ow_ 3.8 *versus* 3.6). The Henry’s law constant for α-HCH is about twice as high as that of γ-HCH, so α-HCH is more likely to partition to the air. Another important difference between the isomers is the persistence of the β-isomer. β-HCH is more resistant to environmental degradation, it is also more lipophilic than the other isomers. These properties may result from its significantly smaller molecular volume. Since bonds between H, C, and Cl in the β-HCH at all six positions are equatorial, the molecule is denser and small enough to be stored in the interstices of lipids in animal tissues.

When comparing HCH isomers with other OPs, HCH exhibit relatively higher water solubility and moderately higher vapor pressures. Therefore, HCH is usually present in the environment as a gas in the atmosphere or dissolved in water, and a small percentage is adsorbed onto particles [59]. Brubaker and Hites [60] measured the gas phase reaction kinetics of the hydroxyl radical with α-HCH and γ-HCH and showed that these compounds have fairly long lifetimes in air and therefore can be transported long distances. Despite these approaches, there is controversy in the literature over the persistence of HCHs in soil, water and air, probably due to contrasting data resulting from the complex interactions of environmental factors affecting rates of both abiotic and biotic removal [37].

All the HCH isomers are acutely toxic to mammals. In addition, chronic exposure has been linked to a range of health effects in humans, including immunosuppression and neurological problems, and has been shown to cause liver cancer in rats and mice [12]; HCHs primarily affect the central nervous system. However, among different isomers, α-HCH and β-HCH are classified as probable human carcinogen and possible human carcinogen (respectively) by the United States Environmental Protection Agency [61]. As the most metabolically stable isomer, β-HCH is the predominant isomer accumulating in human tissues. This tendency to bioaccumulate is a cause of concern as some studies indicate that β-HCH may act as an environmental estrogen [12].

## 3. Biodegradation Pathways of Hexachlorocyclohexane

Different aerobic and anaerobic bacteria have been found to be capable of using halogenated compounds as a growth substrate. Most of the HCH-bioremediating aerobic bacteria and the isolation countries are: *Micromonospora* sp. and several *Streptomyces* spp. strains (Argentina) (see Table 1); *Bacillus* sp. [62] and *Pseudomonas* sp. [63] (Canada); *Sphingobium* sp. MI1205 [64] (China); *Rhodanobacter lindaniclasticus* RP5557 [65,66] and *Sphingobium francense* sp. [67] (France); *Alcaligenes faecalis* S-1, *Alcaligenes faecalis* S-2 [68], *Arthrobacter citreus* BI-100, *Bacillus circulans*, *Bacillus brevis*[69], *Microbacterium* sp. ITRC1 [56], *Pseudomonas aeruginosa*[70], *Pseudomonas aeruginosa* ITRC-5 [71], *Pseudomonas* sp. [72], *Sphingobium chinhatense* IP26 [73], *Sphingobium indicum* B90A [74], *Sphingobium quisquiliarum* P25 [75], *Sphingobium ummariense* RL-3 [76], *Sphingobium* sp. UM2, *Sphingobium* sp. HDU05, *Sphingobium* sp. HDIP04, *Sphingobium* sp. F2, *Sphingomonas* sp. UM1 [77] and *Xanthomonas* sp. ICH12 [78] (India); *Sphingobium japonicum* UT26 [79], *Sphingobium* sp. BHC-A [80], *Sphingobium* sp. SS04-1, *Sphingobium* sp. SS04-2, *Sphingobium* sp. SS04-3, *Sphingobium* sp. SS04-4, and *Sphingobium* sp. SS04-5 [81] (Japan); *Sphingomonas* sp. DS2, *Sphingomonas* sp. DS2-2, *Sphingomonas* sp. DS3–1 [35], *Sphingomonas* sp. α1–2, *Sphingomonas* sp. α4-2, *Sphingomonas* sp. α4-5, *Sphingomonas* sp. α16–10, *Sphingomonas* sp. α16–12, *Sphingomonas* sp. γ1-7, *Sphingomonas* sp. γ12-7, *Sphingomonas* sp. γ16-1 and *Sphingomonas* sp. γ16-9 [36] (Spain) and *Escherichia coli*[82] and *Pseudomonas putida*[83] (USA).

It is important to note that bioremediation involves the intervention aimed at alleviating pollution, where the organism removes the pollutant by different mechanisms (bioaccumulation, biosorption, biodegradation). This section deals only with bacterial biodegradation of HCH, which tackles the biological bases of the metabolism of recalcitrant compounds [30].

### 3.1. Aerobic Hexachlorocyclohexane Degradation in Gram-Negative Microorganisms

The key reaction during microbial degradation of halogenated compounds is the removal of the halogen atom. During this step, the halogen atoms, which are usually responsible for the toxic and xenobiotic character of the compound is most commonly replaced by hydrogen or a hydroxyl group. Halogen removal reduces both recalcitrance to biodegradation and the risk of forming toxic intermediates during subsequent metabolic steps.

Most of the HCH-degrading aerobes known to date are members of the family Sphingomonadaceae [84,85]. Particularly, the research has focused on strains named *Sphingobium japonicum* UT26, *Sphingobium indicum* B90A, and *Sphingobium francense* Sp+ [35,86].

Genes encoding the γ-HCH degradation enzymes have been cloned, sequenced and characterized [78]. These genes (called *lin* genes) were initially identified and characterized for *Sphingobium japonicum* UT26 [87]. Because plasmids were found to be associated with the horizontal gene transfer of *lin* genes among the degraders [67], very similar *lin* genes have also been identified for all the other HCH degrading sphingomonads tested [35,67,80,81,84,88]. Thus, all the degraders isolated from far off geographical locations have a nearly identical set of genes [84] In *Sphingobium japonicum* UT26 [86], the pathway is comprised as follows: *linA*, encoding a dehydrochlorinase [89]; *linB*, encoding a haloalkane dehalogenase [90]; *linC*, encoding a dehydrogenase [91]; *linD*, encoding a reductive dechlorinase [92]; *linE*/*linEb*, encoding a ring cleavage oxygenase [93]; *linF*, encoding a maleylacetate reductase [93]; *linGH*, encoding an acyl-CoA transferase [86]; and *linJ*, encoding a thiolase [86], plus *linR*/*linI*, which are regulatory genes [86,94].

There is evidence that LinA dehydrochlorinates γ, α and δ-HCH, while LinB hydrolytically dechlorinates β and δ-HCH in all strains examined. However, there are strain differences with respect to the hydrolytic dechlorinase-led pathway for β- and δ-HCH. For instance, Datta *et al.*[69] found that γ-HCH aerobic degradation by *Arthrobacter citreus* BI-100 does not present the formation of tetrachloro-cyclohexadiene (1,3,4,6-TCDN) as a transient product by dehydrochlorination of γ-pentachloro-cyclohexene (γ-PCCH) that occurs during the metabolism of γ-HCH by *S. japonicum* UT26 [91]. It is interesting to note the formation of trichlorocyclohexa-diene (TCCD) by *A. citreus* BI-100, in contrast to the production of 1,2,4-trichlorobenzene (1,2,4-TCB), as a dead-end product by *S. japonicum* UT26 during the metabolism of γ-HCH [91]. Furthermore, *S. japonicum* UT26 pathway does not show the appearance of 2-chlorophenol, catechol, and phenol which are present in *A. citreus* BI-100 γ-HCH degradation pathway. The study of Datta *et al.*[69] concludes that some γ-HCH metabolites produced by *A. citreus* BI-100 are quite different from those produced from γ-HCH by any other single microorganism reported in the literature.

On the other hand, Manickam *et al.*[78] isolated a *Xanthomonas* sp. ICH12 strain, capable of biodegrading γ-HCH. During the degradation of γ-HCH by *Xanthomonas* sp. ICH12, the formation of two intermediates, γ-PCCH and 2,5-dichlorobenzoquinone (2,5-DCBQ), was identified. While γ-PCCH was reported previously by *S. japonicum* UT26, 2,5-dichlorohydroquinone was a novel metabolite from HCH degradation.

### 3.2. Aerobic Hexachlorocyclohexane Degradation in Gram-Positive Microorganisms

While there have been many reports regarding aerobic degradation of HCH by Gram-negative bacteria, little information is available on the ability of biodegradation of HCH by Gram-positive microorganisms [13]. This preponderance of data on Gram-negative bacteria reflects the fact that molecular techniques for this group of bacteria are much more advanced when compared with Gram-positive bacteria, rather than the intrinsic metabolic potentials of the respective groups. Regarding this approach, the metabolic pathway for pesticide degradation by actinobacterias has not been studied extensively; however, it is known that these microorganisms can produce extracellular enzymes that degrade a wide range of complex organic compounds. A common feature of the aerobic actinobacterias is the presence of many types of monooxygenases and dioxygenases [95].

Manickam *et al.*[11] isolated the actinobacteria *Microbacterium* sp. strain ITRC1 that has the ability to degrade all four major isomers of HCH. DNA fragments corresponding to the two initial genes involved in γ-HCH degradative pathway, encoding enzymes LinB and LinC, were amplified by PCR and sequenced showing that the two genes present in *Microbacterium* sp. ITRC1 were homologous to those present in *S. japonicum* UT26. Cuozzo *et al.*[46] demonstrated for the first time a specific dechlorinase activity in *Streptomyces* sp. M7 isolated from wastewater sediments of a copper filter plant (Table 1). Using γ-HCH as a specific substrate, the authors demonstrated that synthesis of the dechlorinase enzyme was induced by the presence of the pesticide in the culture medium. This enzyme can function at alkaline pH, whereas dechlorinase activity of *S. japonicum* UT26 was optimal in acidic pH conditions [96]. In addition, the first two metabolites: γ-PCCH and 1,3,4,6-TCDN, produced by the action of dechlorinase over γ-HCH were detected in the cell-free extract of *Streptomyces* sp. M7; both metabolites increased after 96 h of growth. When this strain was cultured in minimal medium with γ-HCH, a differential protein band corresponding to polynucleotide phosphorylase appeared in SDS-PAGE. This enzyme plays an important role in the regulation system and could be involved in the regulation of the dechlorinase gene. Andrade and Arraiano [97] found that the polynucleotide phosphorylase in *Escherichia coli* plays a key role in the regulation of small RNA molecules that control the expression of outer membrane proteins. This enzyme would probably be implied in the regulation of the dechlorinase synthesis in *Streptomyces* sp. M7. On the other hand, Normand *et al.*[98] detected a putative 2,5-dichloro-2,5cyclohexadiene-1,4-diol dehydrogenase (2,5-DDOL dehydrogenase) in *Frankia*, another genus of actinobacteria.

It is important to note that recently, Isaza *et al.*[100] identify a haloalkane dehalogenasa (LinB) belonging to *Mycobacterium tuberculosis* CDC1551. This protein of 300 aminoacids, showed 100% homology to *Mycobacterium tuberculosis* KZN 605, *Mycobacterium bobis* BCG STR Pasteur 117 and 98% homology to *Sphingobiun francensis* and *Sphingobiun* sp. SSD4-1, which would indicate the stability of the gene in Gram-positive as well as in Gram-negative bacteria.

#### 3.2.1. Indirect Assay to Detect Hexachlorocyclohexane Degradation

As mentioned above, biodegradation tackles the biological bases of the metabolism of recalcitrant compounds, which deal with the determination of parent chlorinated compounds as well as their intermediate. Chromatography coupled to mass spectrometry has become a significant tool for elucidating pesticide degradation pathways [11,51,78,101–103]. However, this chromatographic technique requires extraction and cleanup of samples, which implies expensive instrumentation.

Another possibility to detect HCH-degrading activities involves a colorimetric assay that detects chloride released from HCH by dehalogenase enzymes [96]. Since the molecule of HCH has six chlorine atoms, dechlorination is a very significant step in its degradation process. This technique is sensitive, rapid, inexpensive and highly useful for routine screening of a large number of samples simultaneously [96]. Fuentes *et al.*[5] used the colorimetric assay to characterize *Streptomyces* spp. and *Micromonospora* sp. strains (Table 1) able to dechlorinate γ-HCH, chlordane and methoxychlor from the culture medium. Those strains were isolated from soil samples collected from a village named Argentina in the province of Santiago del Estero, (Argentina), where more than 30 tons of obsolete OPs were found in 1994. The authors quantify pesticides removal (by gas chromatography) and indirectly probe its biodegradation by release of chloride ions. Twelve out of 18 studied actinobacteria released chloride to culture medium, and percentages were higher with chlordane as carbon source than with γ-HCH or methoxychlor.

In addition to provide a rapid screening tool for the detection of γ-HCH-degrading microorganisms, this technique was used also to select microbial consortia for γ-HCH degradation. Mixed cultures are considered to be potential agents in biodegradation of recalcitrant compounds because, in some cases, they have proven to be more efficient than pure cultures [48]. However, selecting the best mixed culture usually implies a large volume of work due to the numerous combinations of pure cultures to be performed to choose the best combination for HCH degradation. Fuentes *et al.*[48] measured dechlorinase activity in cell-free extracts of 57 mixed cultures of *Streptomyces* spp. using this technique. The authors found that the combination of four strains consisting of *Streptomyces* spp. A2, A5, A11 and M7 (Table 1) presented the lowest value for the ratio between residual γ-HCH and specific dechlorinase activity, and therefore could be a promising consortium for γ-HCH biodegradation.

## 4. γ-Hexachlorocyclohexane Removal by Actinobacteria

Actinobacteria (Gram-positive bacteria with high G+C content of DNA) are morphologically complex bacteria that represent an important component of microbial soil biodiversity. Depending on the taxon, they may produce branched rods, complex mycelial structures and spore bodies, motile or nonmotile rods. The group has a great potential for bioremediation of organic and inorganic toxic compounds [104] and particularly pesticide-degrading strains are not restricted to a particular genus or family. A review by De Schrijver and De Mot [31] showed that the genera *Arthrobacter*, *Brevibacterium*, *Clavibacter*, *Corynebacterium*, *Mycobacterium*, *Nocardia*, *Rhodococus* and *Micromonospora* are pesticide degrading actinobacterias. However, in that study, only one strain belonging to the *Streptomyces* genus [105] is mentioned as able to dechlorinate γ-HCH. Most recent studies have isolated and characterized *Streptomyces* spp. strains able of degrading OPs (including γ-HCH) and some strains are proposed to be used for soil decontamination (Table 1).

**Figure 2:**
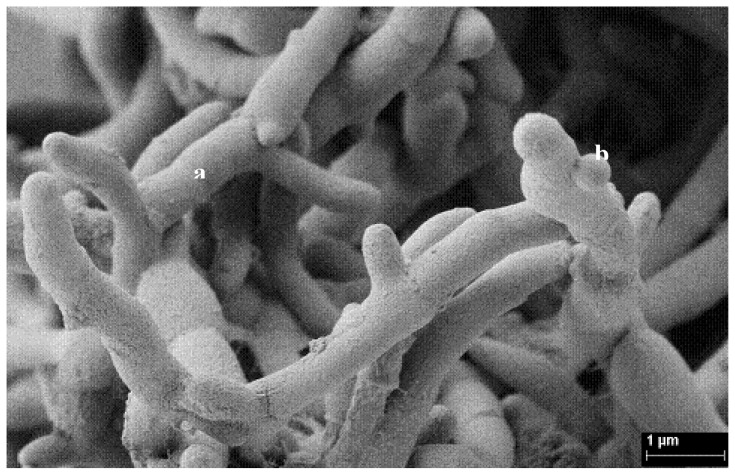
Characteristic vegetative hyphal mass and spores of the *Streptomyces* genus. (**a**) Hyphae; (**b**) Spore forming.

*Streptomyces* genus is well known for their distinct impacts on mankind since its species are prolific producers of antibiotics [106]. In addition to their potential metabolic diversity, *Streptomyces* spp. may be well suited for soil inoculation as a consequence of their mycelial growth habit (Figure 2), relatively rapid rates of growth, colonization of semiselective substrates and their ability to be genetically manipulated [107]. For soil bioremediation purposes, one additional advantage is that the vegetative hyphal mass of these microorganisms can differentiate into spores that assist in spreading and persisting. This can be seen as adaptation to living in soil, especially to extreme environmental conditions such as scant nutrients, intense salt load and low pH, as well as contamination [104].

### 4.1. Culture Condition for γ-Hexachlorocyclohexane Removal in Culture Medium

The fate of organic pollutants in the environment is influenced by environmental factors, such as pH and temperature, affecting the activity of microorganisms and pesticide removal. Benimeli *et al.*[41] Benimeli [99] and Fuentes *et al.*[5] isolated and selected wild type *Streptomyces* spp. strains which were able to tolerate and remove γ-HCH and other OPs (Table 1). In another work, Benimeli *et al.*[44] studied the growth of one of these strains named *Streptomyces* sp. M7 in a poor culture medium (soil extract medium) spiked with γ-HCH, at different temperature and pH values. Despite the poor organic matter, *Streptomyces* sp. M7 was able to grow for limited time. The authors found that the presence of γ-HCH did not inhibit microbial growth, since significant differences in bacterial growth with and without the pesticide were not observed. Similar results were obtained previously [43], when *Streptomyces* sp. M7 was cultured in minimal medium supplemented with γ-HCH 100 μg L^−1^, suggesting that the pesticide could not be toxic for this strain and it would not be either accumulated toxic intermediary metabolites that had an inhibiting effect on the growth. When the effect of the temperature (25 °C, 30 °C and 35 °C) on the growth and pesticide removal of *Streptomyces* sp. M7 was analyzed, it was observed that 25 °C was the optimal temperature for microbial growth. On the other the hand, maximum pesticide removal (70%) was reached at 30 °C. Similar findings were reported by other authors, although they were working with other microorganisms. For instance, Arisoy and Kolankaya [108] observed that the suitable incubation temperature for maximum growth and degradation activity of γ-HCH by the fungus *Pleurotus sajor-caju* was 30 °C. Bachmann *et al.*[109] reported that temperature of 30 °C was most favorable for the biodegradation of γ-HCH in soil slurry system by the mixed native microbial population of the soil. Manonmani *et al.*[110] observed the degradation of the γ-HCH by a microbial consortium under a wide range of temperatures (4 °C–40 °C) in a liquid culture medium. The optimum for γ-HCH degradation was also 30 °C. Siddique *et al.*[111] obtained similar results studying the effect of incubation temperature in the biodegradation of γ-HCH by *Pandoraea* sp.; an incubation temperature of 30 °C was optimum for degradation of γ-HCH (57%) in liquid culture and soil slurry (52%).

Although evidence indicates that the optimum temperature of γ-HCH removal would be 30 °C, Zheng *et al.*[112] demonstrated that haloalkane dehalogenases in *Sphingobium indicum* B90A and *Sphingobium japonicum* UT26 are very active at temperatures as low as 4 °C. Authors who concluded that *Sphingobium* strains might be good candidates for developing novel bioremediation techniques for cold regions, where bioremediation is often limited by lower microbial or enzyme activity induced by low temperatures [43], also studied the behavior of *Streptomyces* sp. M7 cultured with γ-HCH at different initial pH (5, 7 and 9). Pesticide removal (~47% and 38%) was observed at initial pH of 5 and 9, respectively. However, the highest removal ability (~70%) and maximum growth were reached at an initial pH = 7, at 28 days of incubation, which demonstrated that *Streptomyces* sp. M7 was able to remove γ-HCH over a wide range of pH in soil extract medium. Other microorganisms may remove γ-HCH at another optimum pH values. For instance, Arisoy and Kolankaya [108] reported that medium pH = 5 was the optimum for both growth and degradation activity of γ-HCH by the fungus *Pleurotus sajor-caju*. Manonmani *et al.*[110] examined the influence of pH on the degradation of the γ-HCH isomer in a basal mineral medium by an acclimated consortium of microorganisms. They found that a pH range of 6–8 was most favorable for growth and degradation of the pesticide. Siddique *et al.*[111] reported that *Pandoreae* spp. showed the highest degradation of γ-HCH at an initial pH of 8 in broth culture.

### 4.2. Removal of γ-Hexachlorocyclohexane in Soil

Following entry into the soil environment, pollutants rapidly bind to the mineral and organic matter via a combination of physical and chemical processes. Sorption, complexation and precipitation constitute the pollutant-soil interaction. The ability of soils to release pollutants determines its susceptibility to microbial degradation, thereby influencing effectiveness of the bioremediation process [113]. For instance, different soil types can affect redox potential under the same treatment conditions, and these differences in pH and organic matter might contribute to differences in HCH removal rates [37].

In spite of the complexity of the soil system, research about soil bioremediation are the focus of interest, because this kind of studies usually involve treating the polluted material at the site, resulting in a low-cost, low-maintenance, environmentally friendly and sustainable approach for the cleanup of polluted soils.

In a 70-day study, Rodriguez and Toranzos [114] found that the diversity of the overall bacterial cell number in a tropical soil with a history of HCH contamination and spiked with 100 mg kg^−1^ γ-HCH was reduced by 50% relative to the nonspiked control. Benimeli *et al.*[45] found opposite results when studying γ-HCH bioremediation ability of *Streptomyces* sp. M7 growing on sterile soil samples by adding different γ-HCH concentrations. Results showed that significant differences in the growth of *Streptomyces* sp. M7 in soil were not observed at different pesticide concentrations (100, 150, 200 and 300 mg kg^−1^). These contrasting results are not surprising considering that HCH may also affect soil microbial populations, stimulating growth of certain microorganisms and exerting toxic effects and inhibiting the growth of others [37].

On the other hand, a significant influence on biodegradation of δ-HCH could be their concentration in the contaminated soils. There is evidence that pesticide biodegradation rates in soil are concentration dependent [37]. Benimeli *et al.*[45] found that δ-HCH removal by *Streptomyces* sp. M7 reached approximately 29%, 78%, 39% and 14%, at initial δ-HCH concentrations of 100, 150, 200 and 300 μg kg^−1^ (respectively) after four-week incubation. Considering that soil contains readily available organic nutrients that microorganisms may prefer for growing, and as *Streptomyces* sp. M7 growth was continued despite γ-HCH concentration, it would be possible that the pesticide could be used as a secondary substrate source (cometabolism). It is probably the most widespread mechanism for pesticide degradation [31]. This was previously demonstrated by Benimeli *et al.*[43] in culture medium, where glucose at limited concentration was added.

As mentioned above, the use of microbial mixed cultures increases the number of catabolic pathways available to degrade the contaminants [32]. Regarding this approach, Fuentes *et al.*[48] studied the role of three mixed cultures consisting of two, three and four *Streptomyces* spp. strains in decontamination of soil samples polluted with 1.66 mg kg^−1^ γ-HCH. All three consortia exhibited excellent bacterial growth when they were inoculated in γ-HCH-polluted soil and, interestingly, no growth inhibition was observed. An explanation could be the fact that the original environment where the strains were isolated from was highly polluted with OPs (Table 1). Chromatography analysis demonstrated that the three consortia were able to remove about 32% of the pesticide after 28 days of incubation.

## 5. Hexachlorocyclohexane Removal by Plants Interacting with Microorganisms

Some phytoremediation techniques, based on the interactions between plants and their associated microorganisms have been proposed as cost-efficient and eco-friendly methods to clean up polluted soils [38,55]. Microbe assisted phytoremediation appears to be particularly effective for organic pollutants, including the more recalcitrant POPs like HCH [115].

Recent studies demonstrate enhanced dissipation and/or mineralization of OPs at the root-soil interface [38]. This rhizosphere effect is generally attributed to an increase in microbial density and/or metabolic activity due to the release of plant root exudates (REs). REs contain water soluble, insoluble, and volatile compounds, including sugars, amino acids, organic acids, nucleotides, flavonoids, phenolic compounds and certain enzymes [116]. Since REs are complex mixtures of substrates, they not only provide a nutrient-rich habitat for pollutant degraders but can also potentially enhance biodegradation in different ways: they may facilitate the cometabolic transformation of pollutants with similar structures, induce catabolic enzymes involved in the degradation process and/or enhance the contaminant bioavailability [38,117]. In addition, REs may directly induce contaminant degradation by root-driven extracellular enzymes [117,118] as was demonstrated by Magee *et al.*[119], who reported dechlorination of polychlorinated biphenyls (PCBs) by crude extract of nitrate reductase from *Medicago sativa* and a pure commercial nitrate reductase from maize. Similar results were obtained by Alvarez *et al.*[54], who detected specific dechlorinase activity (SDA) in maize REs. In that study, SDA explained 42% of γ-HCH degradation in minimal medium supplemented with REs and γ-HCH.

Phytostimulation of OP-degrading microorganisms is therefore likely to be a successful strategy for the remediation of γ-HCH-contaminated environments. However, a careful selection of tolerant plant species and optimizing plant growth are vital since the phytotoxic nature of these contaminants can inhibit plant performance, reducing the overall efficiency of the remediation process [55,120,121]. Some plants are well adapted to acidic conditions as generated during OPs degradation. For instance, Benimeli *et al.*[45] showed that γ-HCH concentration of 100, 200 and 400 μg kg^−1^ soil did not affect the germination and vigor index of maize plants seeded in contaminated nonsterile soils. Additionally, maize plants may create particularly good environmental condition for soil microorganisms [122]. Likewise, the leguminous species *Cytisus striatus* grow spontaneously on HCH-contaminated sites and has been proposed as a candidate species for the cleanup of this type of contaminant [38,123]. Becerra-Castro *et al.*[55] inoculated substrates seeded with *Cytisus striatus* with the previously isolated bacteria *Rhodococcus erythropolis* ET54b and *Sphingomonas* sp. D4 [55,123]. The authors found that substrates planted with *C. striatus* showed a higher dissipation of HCH isomers and that both microorganisms protected the plants against the toxic effects of the contaminant. Consequently, they propose that inoculating *C. striatus* with this combination of bacterial strains could therefore be a promising approach for the remediation of HCH-contaminated sites. The presence of plant-colonizing, naphthalene-degrading bacteria were shown to protect their host plants against the toxic effects of naphthalene exposure [116,124]. Likewise, the trichloroethylene (TCE)-degrading poplar endophyte *Pseudomonas putida* W619-TCE promoted plant growth and reduced TCE phytotoxicity in inoculated poplar cuttings [121].

Alvarez *et al.*[54] evaluated the effect of maize REs on growth and δ-HCH removal by *Streptomyces* sp. A5 and *Streptomyces* sp. M7 (Table 1). Results showed that both strains were able to grow on minimal medium supplemented with maize REs as sole carbon source, suggesting that these microorganisms are competitive at the rhizosphere level. Furthermore, maize REs markedly influenced the δ-HCH removal by both *Streptomyces* sp. A5 and M7 since pesticide removal reached approximately 55 and 35%, respectively, when REs are present in the culture medium. On the contrary, no decrease in pesticide concentration was observed by Renzt *et al.*[125] and Louvel *et al.*[126], who studied the repression of phenanthrene-degrading activity of *Pseudomonas putida* in the presence of REs of different plant species. The authors suggest that as REs are a complex mixture of substrates; some of them could be repressing microbial-degrading activity.

On the other hand, fungi growing in symbiotic association with plants have unique enzymatic pathways that help to degrade pesticides that could not be transformed solely by bacteria [2]. For instance, mycorrhizal fungi form symbioses with a broad range of plant species and can contribute to plant growth and survival by reducing stresses associated with toxic wastes. The effects of soil HCH contamination on vegetation and its associated arbuscular mycorrhizas was investigated by Sáinz *et al.*[127]. The authors found that a preinoculation of four plant species with an isolate of *Glomus deserticola* obtained from the HCH-contaminated soil resulted in increased growth and fungal colonization of roots, suggesting that the fungus increases the tolerance of plants to the toxic soil environment.

## 6. Conclusions

HCH is one of the most extensively used OPs for both agriculture and medical purposes. Currently, its use is being phased out because of its toxicity, environmental persistence and accumulation in the food chain. Though the use of technical mixture containing four isomers was banned in several advanced countries, many developing countries continue using γ-HCH (lindane) for economic reasons. Thus, new sites are continuously being contaminated by γ-HCH and the other HCH-isomers. Considerable research on chemical pollutants now provides the necessary body of knowledge to understand their recalcitrance and toxic nature. A possible pathway for remediation of HCH-contaminated soils is the use of indigenous bacteria. There have been many reports regarding aerobic degradation of HCH by Gram-negative bacteria, especially those belonging to the *Sphingomonas* genus. The sphingomonads are clearly the group of first choice for further work on strain development for bioremediation purposes, mainly due to the extensive knowledge reached about its metabolic pathways of HCH-degradation. This preponderance of data on Gram-negative bacteria reflects the fact that molecular techniques for this group of bacteria are much more advanced when compared with Gram-positive bacteria, rather than the intrinsic bioremediation potentials of the respective groups. Regarding this approach, Gram-positive bacteria like actinobacteria, are highly promising for HCH-bioremediation purposes. Strains of the *Streptomyces* genus have been seen to present particularly great potential for remediation of toxic organic and inorganic compounds. In fact, most recent studies have isolated and characterized *Streptomyces* spp. able to dechlorinate γ-HCH and some strains are proposed to be used for soil decontamination. Furthermore, phytostimulation of γ-HCH-degrading *Streptomyces* spp. appear to be a successful strategy for the remediation of γ-HCH-contaminated environments.

Work to date indicates that *Streptomyces* spp. have the requisite capabilities to degrade γ-HCH, although further studies on enzymes characterization, particularly for Lin enzymes, are needed to develop effective bioremediation technologies.

## Figures and Tables

**Table 1:** γ-Hexachlorocyclohexane-degrading actinobacteria isolated from Argentina.

Actinobacteria strain	Isolation source	GenBank access number	Action on γ-HCH
*Micromonospora* sp. A10	a OPs contaminated soil	GQ867054 [5]	c Grow, remove and degrade [5,48]
*Streptomyces* sp. A1	OPs contaminated soil	GU085102 [5]	c Grow, remove and degrade [5,48]
*Streptomyces* sp. A2	OPs contaminated soil	GU085103 [5]	c Grow, remove and degrade [5,48]
*Streptomyces* sp. A3	OPs contaminated soil	GU085104 [5]	c Grow, remove and degrade [5,48]
*Streptomyces* sp. A5	OPs contaminated soil	GQ867050 [5]	c Grow, remove and degrade [5,48]
*Streptomyces* sp. A6	OPs contaminated soil	GQ867051 [5]	c Grow, remove and degrade [5,48]
*Streptomyces* sp. A7	OPs contaminated soil	GQ867052 [5]	c Grow, remove and degrade [5,48]
*Streptomyces* sp. A8	OPs contaminated soil	GQ867053 [5]	c Grow, remove and degrade [5,48]
*Streptomyces* sp. A11	OPs contaminated soil	GQ867055 [5]	c Grow, remove and degrade [5,48]
*Streptomyces* sp. A12	OPs contaminated soil	GQ867056 [5]	c Grow, remove and degrade [5,48]
*Streptomyces* sp. A13	OPs contaminated soil	GQ867057 [5]	Grow and remove [5]
*Streptomyces* sp. A14	OPs contaminated soil	GU085105 [5]	c Grow, remove and degrade [5,48]
*Streptomyces* sp. C39	Non-contaminated water	AY741282 [39]	Grow and remove [5]
*Streptomyces* sp. MC1	Sugar cane	AY741287 [39]	Grow (Unpublished data)
*Streptomyces* sp. M7	b Co-contaminated wastewater sediment	AY459531 [43]	d Grow, remove and degrade [5,41–46,48,99]
*Streptomyces* sp. M15	Co-contaminated wastewater sediment	GQ867058 [5]	Grow and remove [5,41]
*Streptomyces* sp. M30	Co-contaminated wastewater sediment	GU085106 [5]	Grow and remove [5]
*Streptomyces* sp. M50	Co-contaminated wastewater sediment	GQ867059 [5]	c Grow, remove and degrade [5]

a Organochlorine pesticides (OPs);

b Wastewater sediments contaminated with Cu and eleven Ops;

c Degradation was determined indirectly by specific dechlorinase activity;

d Degradation was determined by the detection of intermediate metabolites of the catabolic pathway of HCH.
